# Telomere dynamics in free-living edible dormice (*Glis glis*): the impact of hibernation and food supply

**DOI:** 10.1242/jeb.140871

**Published:** 2016-08-15

**Authors:** Franz Hoelzl, Jessica S. Cornils, Steve Smith, Yoshan Moodley, Thomas Ruf

**Affiliations:** Department of Integrative Biology and Evolution, University of Veterinary Medicine, Vienna, Savoyenstraße 1, Vienna 1160, Austria

**Keywords:** Real-time PCR, Feeding experiment, Torpor, Somatic maintenance, Aging

## Abstract

We studied the impact of hibernation and food supply on relative telomere length (RTL), an indicator for aging and somatic maintenance, in free-living edible dormice. Small hibernators such as dormice have ∼50% higher maximum longevity than non-hibernators. Increased longevity could theoretically be due to prolonged torpor directly slowing cellular damage and RTL shortening. However, although mitosis is arrested in mammals at low body temperatures, recent evidence points to accelerated RTL shortening during periodic re-warming (arousal) from torpor. Therefore, we hypothesized that these arousals during hibernation should have a negative effect on RTL. Here, we show that RTL was shortened in all animals over the course of ∼1 year, during which dormice hibernated for 7.5–11.4 months. The rate of periodic arousals, rather than the time spent euthermic during the hibernation season, was the best predictor of RTL shortening. This finding points to negative effects on RTL of the transition from low torpor to high euthermic body temperature and metabolic rate during arousals, possibly because of increased oxidative stress. The animals were, however, able to elongate their telomeres during the active season, when food availability was increased by supplemental feeding in a year of low natural food abundance. We conclude that in addition to their energetic costs, periodic arousals also lead to accelerated cellular damage in terms of RTL shortening. Although dormice are able to counteract and even over-compensate for the negative effects of hibernation, restoration of RTL appears to be energetically costly.

## Abstract (precis)

**Highlighted Article:** Telomeres in edible dormice shorten over the hibernation season, but these long-lived rodents are able to fully restore telomeres during summer if food supply is sufficient.

## INTRODUCTION

Hibernators survive the winter season surprisingly well (97% monthly survival probability), leading to a 15% higher annual survival probability compared with non-hibernators of similar body size (Turbill et al., 2011). Life history theory predicts that this increased survival probability should select for high investment into somatic repair and maintenance (Austad and Fischer, 1991; Kirkwood and Austad, 2000; Lyman et al., 1981; Turbill et al., 2011). One marker of somatic maintenance and damage is the length of telomeres, the endcaps of chromosomes in eukaryotic cells (Meyne et al., 1989). Together with telomere-associated proteins (the shelterin complex), telomeres prevent the degradation of linear DNA, but shorten with every cell division – because of the end replication problem in mitosis (Blackburn, 1991; Blasco, 2007). In addition to the shortening during cell proliferation, oxidative stress has a strong effect on telomere erosion (Proctor and Kirkwood, 2002; von Zglinicki, 2002). Although the relationship with longevity is not universal or entirely clear, telomere length and, in particular, the rate of telomere shortening often correlate with aging rates and future survival (Boonekamp et al., 2014; Monaghan, 2010; Salomons et al., 2009).

Because mitosis is arrested at low body temperature (*T*_b_) during hibernation in the G2 stage (Kruman et al., 1988), further telomere shortening, which only occurs during cell division, should be prevented by deep torpor (Marcand et al., 2000). However, periodic rewarming during the hibernation season is associated with accelerated mitotic activity (Kruman et al., 1988; Vinogradova, 1988) and high production of reactive oxygen species (ROS) (Buzadžić et al., 1990; Carey et al., 2000; Orr et al., 2009). Not surprisingly then, Turbill et al. (2013) found that RTL shortening in hibernating edible dormice was associated with body mass loss, which itself is proportional to the time spent euthermic during hibernation (i.e. at high *T*_b_ during intermittent arousals from torpor). Furthermore, Giroud et al. (2014) found that in hibernating garden dormice the degree of relative telomere length (RTL) shortening over a 3-month period was correlated with the total time the animals spent euthermic. Neither of these studies was designed, however, to clarify whether RTL loss is indeed mainly determined by the time hibernators remain euthermic in winter, or rather by the frequency of rewarming from torpor, as thermogenesis during arousal seems particularly associated with oxidative stress (Orr et al., 2009).

The edible dormouse is a particularly suitable model with which to study this question as the total duration of hibernation and associated traits (namely, the frequency of arousals and the total time spent at euthermia) are highly variable (Bieber et al., 2014; Bieber and Ruf, 2009; Hoelzl et al., 2015). This variability is linked to strong responses of dormice to the availability of high-caloric seeds (e.g. European beech, *Fagus sylvatica*) in summer and autumn, a crucial factor in the animals' life history determining the probability of reproduction as well as the onset of hibernation (Hoelzl et al., 2015). Beech trees are particularly important for juveniles to gain sufficient body fat reserves prior to hibernation. Therefore, in years without sufficient amounts of beechnuts, the animals skip reproduction because of the low probability of juvenile survival (Bieber, 1998; Lebl et al., 2010; Ruf et al., 2006; Schlund et al., 2002) and they may start hibernation extremely early (i.e. July; Hoelzl et al., 2015). However, dormice can show this very early onset of hibernation only if they can afford it energetically, that is, if body energy reserves are high (Hoelzl et al., 2015). This leads to a large variation in the use and characteristics of prolonged torpor in this species, which provides the opportunity to investigate the effects of differences in individual hibernation patterns on RTL change.

We hypothesized that RTL shortening should be most pronounced in animals with a high frequency of rewarming phases and/or longer times spent euthermic during arousals within the hibernation season. Furthermore, we expected that edible dormice would use high food availability during summer to invest incoming energy to some extent into somatic maintenance (i.e. telomere elongation), and possibly to prepare for times of unfavorable conditions. We addressed these questions by determining RTL dynamics, determined in mucosa cells, during the hibernation and the active season in free-living edible dormice in the Vienna Woods. To assess hibernation patterns in detail, we continuously recorded *T*_b_ using intraperitoneally implanted data loggers. To investigate how fluctuating food availability influences telomere dynamics, we conducted a supplemental feeding experiment at the study site.

## MATERIALS AND METHODS

### Ethics statement

All experiments carried out in this study were in accordance with the institutional (University of Veterinary Medicine, Vienna) and national guidelines in Austria according to Section 8ff of the Law for Animal Experiments (Tierversuchsgesetz). The experimental procedures were approved by the Austrian Ministry of Science, Research and Economy (permit no. BMWF-68.205/0112-II/3b/2011).

### Study site

All edible dormice [*Glis glis* (Linnaeus 1766)] were free-living in the Vienna Woods, Austria (48°05′N, 15°54′E; altitude 400–600 m a. s. l.). The study site included approximately 650 ha of deciduous forest dominated by beech (*Fagus sylvatica*, 60%) and spruce (*Picea abies*, 15%). Other tree species occur only in low numbers (for further information about the study site, see Lebl et al., 2011).

Nest boxes (124) positioned at irregular distances (height: 2–3 m) were checked at 2-week intervals. Between April and October (active season of edible dormice), all animals occupying the nest boxes were captured and marked with subcutaneous transponders (BackHome BioTec, Virbac Limited, Bury St Edmunds, UK; Tierchip Dasmann, Greven, Germany) and age was determined by fur color and size at the first capture event (according to criteria given by Schlund et al., 2002). At each capture occasion, dormice were weighed to the nearest 2 g using a 300 g spring balance (Pesola, Baar, Switzerland) and reproductive activity was determined (males: testis size, females: signs of pregnancy or lactation and presence of juveniles).

### Hibernation parameters

**Fig. 1.**
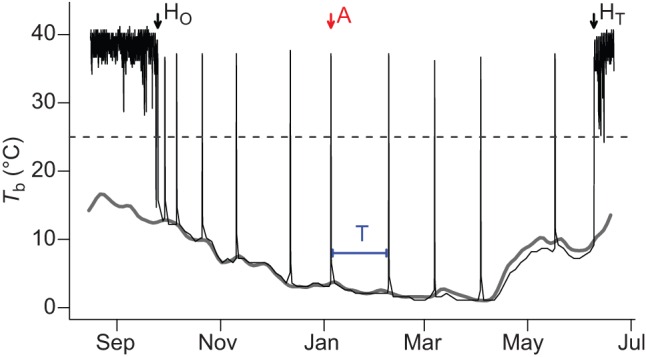
**Records of core body temperature (*T*_b_; solid black lines) in a free-living edible dormouse between August 2012 and July 2013.** Torpor *T*_b_ during hibernation was close to soil temperature (solid gray line). The dashed line indicates the threshold temperature (25°C) used for the computation of times spent torpid and euthermic. H_O_, onset of hibernation; A, arousal; H_T_, termination of hibernation; T, torpor bout.

To assess hibernation characteristics such as number of arousals, time spent euthermic, hibernation duration and time spent torpid, we recorded core body temperature at hourly intervals using intraperitoneally implanted i-buttons (DS1922L, Maxim, Dallas, TX, USA; resolution 0.5°C). Implantation (June and July 2012) and explantation (May to July 2013) of the temperature loggers were carried out under general anesthesia. For detailed description of surgical procedures and anesthesia, see Hoelzl et al. (2015) and Bieber et al. (2014). We recaptured 15 out of 43 implanted and released dormice with complete hibernation periods in 2013, which was similar to the general recapture rate in the population (Hoelzl et al., 2015). For these animals, complete *T*_b_ records were obtained and analyzed. To define the onset and termination of torpor bouts during prolonged torpor, we used a threshold temperature of 25°C. Hibernation onset was defined as the first time point after which *T*_b_ remained below 25°C for at least seven consecutive days. Termination of hibernation was considered to end when *T*_b_ remained >25°C for more than 3 days. Additionally, we computed the number of arousals (number of phases with *T*_b_>25°C), as well as the duration of interbout euthermic phases (‘time spent euthermic’; hours *T*_b_>25°C) and each torpor-bout duration (‘time spent torpid’; hours *T*_b_<25°C; Fig. 1).

Body mass and DNA samples for telomere length analysis were taken at the capture site at the time of implantation and explantation of the temperature loggers.

### Feeding experiment

During the active season in 2014 (a year without reproduction because of low beechnut abundance), we conducted a feeding experiment in part of the population (76 nest boxes), while the remaining part served as the control group (48 nest boxes). The two spatially separated areas did not differ in their habitat characteristics or in demographic population structures. No animal used nest boxes at both sites during the study period. The nest boxes occupied by dormice in the surplus food area were provided with 200 g sunflower seeds every 2 weeks from the end of May to the end of July (six times in total), and DNA samples of the animals were taken at the closest time possible to the beginning and the end of the feeding experiment.

### Telomere length assessment

Cells for DNA extraction were collected from buccal mucosa by twirling Gynobrush brushes (Heinz Herenz Medizinalbedarf, Hamburg, Germany) on the inner cheek for 15 to 20 s. The heads of the brushes were placed into 1.7 ml Mµlti-SafeSeal Tubes (Carl Roth, Karlsruhe, Germany) containing 1 ml BC buffer (Thomas and Fenech, 2008) and stored at 4°C for subsequent DNA extraction, which was always carried out within 24 h of cell collection. The DNA was extracted using the DNeasy Blood & Tissue Kit (Qiagen) according to the manufacturer's protocol after the cells were pelleted and the brushes were removed. For measuring RTL, we used the real-time PCR approach (Thomas et al., 2008) adapted for edible dormice. As the non-variable copy number (non-VCN) gene, we used a 54 bp portion of the c-Myc proto-oncogene, which was tested for non-variability in copy number as described by Cawthon (2002), Smith et al. (2011) and Turbill et al. (2012). Primer sequences for the non-VCN gene were 5′-GAG GGC CAA GTT GGA CAG TG-3′ (c-mycF) and 5′-TTG CGG TTG TTG CTG ATC TG-3′ (c-mycR), and telomeric primer sequences were 5′-CGG TTT GTT TGG GTT TGG GTT TGG GTT TGG GTT TGG GTT-3′ (tel 1b) and 5′-GGC TTG CCT TAC CCT TAC CCT TAC CCT TAC CCT TAC CCT-3′ (tel 2b). Telomere and non-VCN gene PCRs were carried out in separate runs with 20 ng DNA per reaction, 400 nmol l^−1^ of each primer (Tel1b/Tel2b or c-Myc) in a final volume of 20 µl containing 10 µl of SensiMix SYBR No-ROX-MasterMix (Bioline). PCR conditions for the telomere primers were 10 min at 95°C followed by 40 cycles of 10 s at 95°C, 20 s at 56°C and 20 s at 72°C. For c-Myc, PCR conditions were 10 min at 95°C followed by 40 cycles of 10 s at 95°C, 20 s at 61°C and 20 s at 72°C. A final melting step was included in each run with the temperature ramping from 65 to 95°C in 1°C steps. All ratios of telomere to non-VCN gene were compared with a reference standard sample (RTL=1), which was included in every run. A negative (no-template) control was also included in each run. Reactions were prepared using the Qiagility PCR robot (Qiagen, Germany) to minimize pipetting errors, and cycling was performed on a Rotorgene Q quantitative thermocycler (Qiagen, Germany). All samples and controls were run in triplicate. We used the software LinRegPCR (2012.0) (Ramakers et al., 2003; Smith et al., 2011) for analysis of non-baseline-corrected raw qPCR data, exported from the instrument. RTL was calculated using a modified formula described previously in Ruijter et al. (2009):
(1)
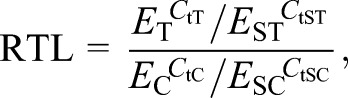


where *E* is the qPCR efficiency, *C*_t_ is the cycle threshold, and the subscript T refers to the telomere reaction of the target sample, ST to the telomere reaction of the standard sample, C to the control gene (c-Myc) reaction of the target sample and SC to the control gene reaction of the standard sample.

Mean qPCR efficiencies were 96.9 and 96.4% for the non-VCN gene and telomere reactions, respectively. The mean coefficient of variation among replicates (intra-assay variation) for *C*_t_ values of the non-VCN gene and telomere assay were 0.39 and 0.67%, respectively. Among runs (inter-assay variation), the mean coefficient of variation for *C*_t_ values of the non-VCN gene was 0.70%, and this was 0.69% for the telomere reaction.

### Statistics

All statistical tests were carried out using R 3.2.1 (R Core Team, 2015). To test for a normal distribution of model residuals, Shapiro–Wilks tests were used. To explain variation between individuals in RTL changes (computed by final RTL as % of initial RTL), linear models were used. Coefficients, their standard errors (s.e.), adjusted *R*^2^, *t* and corresponding *P*-values of the models are reported.

For analyzing hibernation characteristics, sample size was limited to *n*=15 (animals with temperature loggers and complete hibernation records that could be recaptured), so we restricted the number of predictor variables to two per model (initial RTL+one other predictor variable; see Table 1) to avoid over-fitting.

**Table 1.**
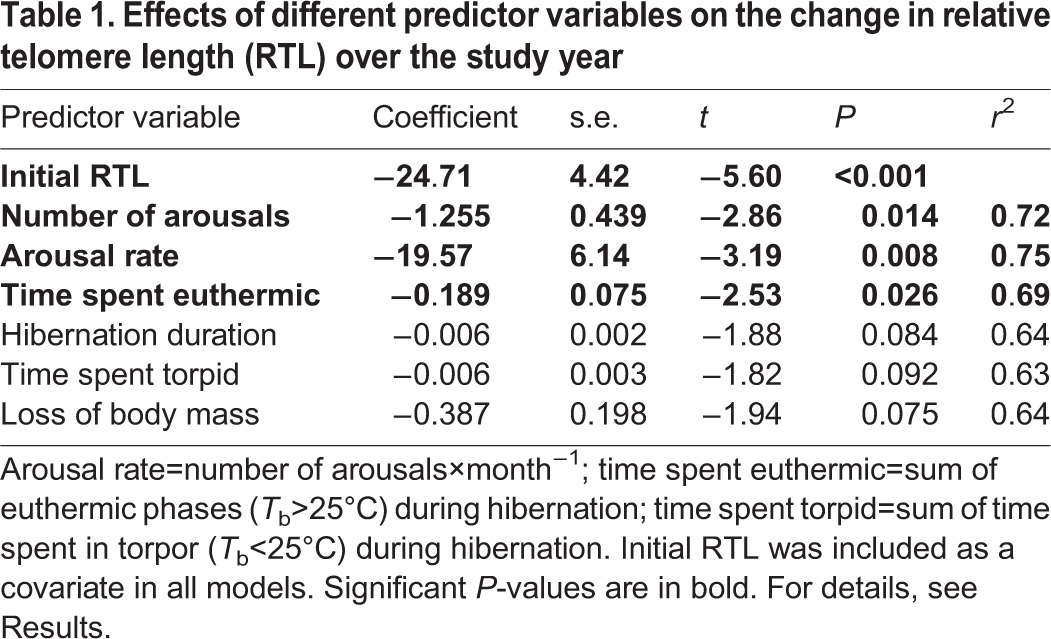
**Effects of different predictor variables on the change in relative telomere length (RTL) over the study year**

For the feeding experiment, the initial model contained initial RTL, sex, age class, change of body mass and timespan between DNA sampling points as predictor variables, and model selection was carried out using the function stepAIC (library MASS; Venables and Ripley, 2002).

A potential problem in the statistical analysis of changes to RTL is the ‘regression to the mean’, i.e. extreme values measured in a subject during a first trial are likely to lie closer to the mean in subsequent trials (Verhulst et al., 2013). Consequently, one would expect stronger apparent shortening of RTL in animals in which initial RTL was high. Further, if RTL shortening was constant, one would expect a negative effect of initial RTL on % RTL reduction and vice versa. Therefore, we used a multiple regression approach, including initial RTL as a covariate in all models, which accounts for these potential effects. The slight variation in the timespan between tissue sampling dates was excluded, as it had no relevant effect (*F*_1,12_=1.79, *P*=0.21). To test for effects of different hibernation parameters (i.e. hibernation duration, arousal rate, total number of arousals, time spent euthermic during the hibernation season, or time spent in torpor) on RTL change, we entered these variables alternatively, rather than simultaneously, into the model (in addition to initial RTL). This was mainly because of the high multicollinearity of the hibernation parameters, and to avoid over-fitting.

All means are given together with their standard error (s.e.m.). For variables repeatedly determined in the same animal (e.g. torpor-bout duration), means were computed from means per individual.

## RESULTS

### Telomeres and hibernation

**Fig. 2.**
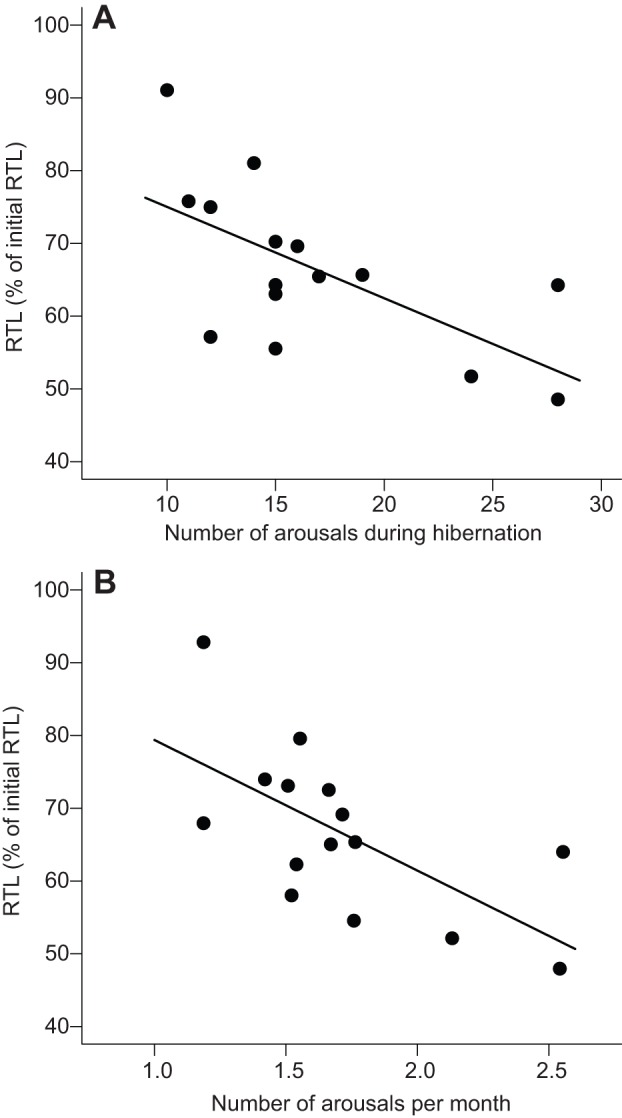
**Impact of arousals on relative telomere length.** Effect of the (A) number of arousals (*R*^2^=0.72) and (B) number of arousals per month (*R*^2^=0.75) during hibernation on relative telomere length (RTL) over approximately 1 year (350±5 days) in 15 edible dormice.

RTL declined in all recorded dormice with implanted i-buttons (*n*=15, mean −33.4±4.6%, range: −4.7 to −58.8%) between the dates of tissue sampling (i.e. early summer 2012 to early summer 2013; mean timespan: 350±5 days). As expected, the degree of RTL shortening was clearly related to initial RTL (Table 1). Among the hibernation characteristics, the arousal rate was the best predictor for the decline in RTL, followed by total number of arousals and time spent euthermic with the hibernation period (Table 1, Fig. 2). Hibernation duration, time spent torpid and loss of body mass had similar apparent effects on the decline of RTL, but to a lesser degree (Table 1). It should be noted that hibernation duration, time spent torpid and loss of body mass were all positively correlated with arousal rate (0.58<*r*<0.74, *P*<0.03). Further, because dormice spent 97.1±0.14% of the hibernation period in torpor, hibernation duration and time spend torpid were almost identical (*r*=0.999).

### Feeding experiment and telomeres

**Fig. 3.**
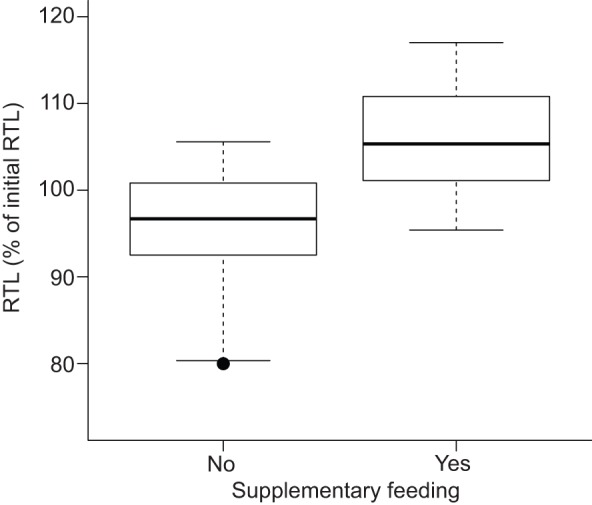
**Effect of supplementary feeding on relative telomere length (RTL) in the early active season (May to July 2014) in 50 edible dormice.**

In total, we quantified the telomeric DNA of 50 animals (supplemented=28, non-supplemented=22) before and after the feeding experiment. The degree of RTL shortening was significantly related to initial RTL (*F*=21.67, *P*<0.001) and the feeding regime (supplementary feeding yes/no; *F*=11.92, *P*<0.01; Fig. 3). Sex, age, change of body mass and timespan between tissue sampling were not included in the best model after model selection.

## DISCUSSION

Our results show that, after correcting for initial telomere length (see Materials and methods), high rates of arousals from torpor had a strongly negative impact on RTL (Fig. 2). This outcome initially seems surprising because hibernators show increased longevity (Austad and Fischer, 1991; Lyman et al., 1981; Turbill et al., 2011). Hence, one might expect that hibernation itself slows down cellular aging processes such as RTL loss. Although this is probably true for the torpid state in which mitosis is arrested (Kruman et al., 1988; Vinogradova, 1988), RTL shortening is evidently accelerated during arousals from torpor (Table 1).

Our finding that RTL shortening also slightly increased as hibernation duration increased (Table 1) can be attributed to the fact that hibernation duration was correlated with arousal rate. This effect occurs because prolonged hibernation extends into the summer, in which higher temperatures within hibernacula lead to increased arousal frequency (Bieber and Ruf, 2009; Hoelzl et al., 2015). Hence, the apparent negative effect of hibernation duration on RTL can also be explained by differences in arousal rates.

Previous results (Giroud et al., 2014; Turbill et al., 2011) already suggested a negative impact of arousals on RTL, but our study is the first to directly investigate the relationship between RTL changes, *T*_b_ patterns and arousal frequencies over the hibernation season in a free-ranging mammal. Interestingly, the rate and frequency of arousals explained more variance in RTL loss than the time spent euthermic during hibernation (Table 1). This supports the hypothesis that phases of rewarming from low *T*_b_ and the sudden upregulation of metabolic rate that seem to be associated with high ROS production (Buzadžić et al., 1990; Carey et al., 2000; Orr et al., 2009), may be particularly detrimental for RTL. Hence, although hibernation helps to minimize mortality (e.g. by reduced predation; Bieber and Ruf, 2009; Geiser and Brigham, 2012; Turbill et al., 2011), which should increase the beneficial effects of intensified investment into somatic maintenance (Kirkwood, 1977; Kirkwood and Austad, 2000), its immediate net effect is RTL shortening. This acceleration of cellular aging is especially notable if hibernation is extended into warmer months, which is associated with a higher rate of arousals. Further, our finding that RTL shortening was most pronounced in animals that had long initial RTL (Table 1) could indicate that larger telomeres are more susceptible to oxidative stress during arousals than shorter telomeres, but this requires further investigation.

It is still unclear why hibernators arouse and invest large amounts of body energy reserves into repeated rewarming during hibernation. Different physiological functions, such as sleep (Daan et al., 1991; Kräuchi and Deboer, 2011), immunocompetence (Prendergast et al., 2002), memory (Millesi et al., 2001) or long-term cardiac function (Ruf and Arnold, 2008), have been suggested to be adversely affected by deep torpor and may limit the time hibernators can continuously remain in this state. Interestingly, for the same duration of the hibernation season, dormice rewarm more often if their body mass (i.e. fat reserves) at hibernation onset is high (Bieber et al., 2014). This has also been shown in other hibernating species such as woodchucks (*Marmota monax*) (Zervanos et al., 2014). This indicates that hibernators, if body energy reserves permit them to do so, actively minimize the putative harmful effects of deep torpor mentioned above (see Humphries et al., 2003) despite the adverse consequences of increased arousal frequency on RTL. Hibernating small rodents may tolerate telomere shortening because (unlike in most human tissues) it can be reversed by the enzyme telomerase (Greider and Blackburn, 1985) or alternative lengthening of telomeres (ALT) (Neumann et al., 2013), which can cause telomere elongation. As telomere dynamics and high telomerase activity are also associated with cancer (Blasco, 2005; Kim et al., 1994), it is important to note that we have never detected any evidence of tumors in our study population of edible dormice.

Interestingly, it seems that telomere dynamics in hibernators that reach *T*_b_ values in torpor just above ambient temperature differ from those in daily heterotherms, which maintain much higher torpor *T*_b_ values. Whereas return to euthermia was associated with telomere shortening in hibernating edible dormice and garden dormice (present results; Giroud et al., 2014; Turbill et al., 2013), the frequent use of daily torpor had a positive effect on RTL in Djungarian hamsters (Turbill et al., 2012). This difference could mean that in daily heterotherms, torpor has an RTL-conserving effect (because of absent or attenuated mitosis), while rewarming from relatively high *T*_b_ (∼20°C) may not lead to oxidative stress levels that cause telomere damage. If telomerase activity remains constant, this should result in a net increase in RTL as the frequency of daily torpor increases, as observed by Turbill et al. (2011). Clearly, however, more studies on other species are needed to test this explanation.

Turbill et al. (2013) have previously shown that in edible dormice kept under *ad libitum* food supply, telomeres can be elongated in summer, but it remained unclear which factors caused an increase of RTL. Our present results show that re-elongation of telomeres during the active season requires high food availability (Fig. 3). This finding is slightly puzzling because studies on laboratory rodents showed that caloric restriction had a positive effect on telomere length (Masoro, 2005; Weiss and Fontana, 2011) and so it seems surprising that dormice showed telomere elongation when food availability was artificially elevated. This effect could be explained by the fact that free-living animals under natural food shortage have to increase their foraging effort to supply the organism with a sufficient amount of energy. Enhanced foraging behavior is associated with higher predation risk, which can lead to increased production of stress proteins, decreased antioxidant defenses and elevated oxidative stress (Slos and Stoks, 2008). Further, high levels of physical activity that may occur during arboreal foraging of dormice can also increase oxidative stress (Ludlow et al., 2012, 2008). High oxidative stress levels in turn negatively affect telomere integrity (Beaulieu et al., 2010; Costantini et al., 2010; Monaghan et al., 2009).

Our results indicate that, if food availability is high, as is the case in years in which beech and oak are seeding, dormice do not just compensate for RTL loss during the hibernation season, but elongate telomeres beyond their initial length in the previous year (Fig. 3). This suggests that the animals may use telomere restoration in a manner similar to fat storage for unfavorable conditions. Hence, an anticipatory elongation of telomeres could represent a mechanism to ensure that cells can keep their mitotic abilities by avoiding the likelihood of cell senescence during and immediately after hibernation.

However, recent data on long-term telomere dynamics (F.H., S.S., J.S.C., D. Aydinonat, C. Bieber and T.R., unpublished data) indicate that edible dormice do not just maintain constant RTL, but in fact increase RTL with progressing age. This elongation of telomeres was paralleled by a significant increase in the probability of reproduction in older females. Therefore, telomere elongation could also represent a preparation for upcoming reproduction events. As 2014 was a mast failure year and no successful reproduction was detected, reproduction was not included in the analyses of the present study. However, female dormice show very high rates of energy turnover (Ruf et al., 2006) and juveniles grow extremely fast (Bieber and Ruf, 2004) because of the short time period of highly abundant food in late summer. Therefore, reproduction-related oxidative stress and the concomitant telomere erosion seem likely in this species, and reproduction may well play an important role in the telomere dynamics of edible dormice. Given that reproduction in this species is restricted to years with high food abundance (Bieber, 1998; Lebl et al., 2010; Pilastro et al., 2003; Ruf et al., 2006), our finding of a positive effect of surplus food on RTL suggests a pathway by which preemptive telomere elongation and reproduction may be physiologically linked.

Taken together, our data reveal that hibernation, and in particular periodic arousals, has a strong negative impact on telomere length in free-living edible dormice. However, the animals are capable of counteracting and even over-compensating for this effect if food availability is sufficient. Our data also suggest that under natural conditions, increased investment into somatic maintenance, at least in terms of telomere elongation, does not occur during the hibernation season. This may indicate that RTL re-elongation cannot occur during euthermic phases during the hibernation season, either because RTL shortening continues during these phases or because telomerase or ALT activity is energetically too costly to rely on body energy reserves alone. We cannot rule out, however, that telomere elongation mechanisms are active during arousals but are insufficient to compensate for the oxidative damage occurring during the rewarming phases. At any rate, the actual energetic costs of telomere elongation are unknown, but probably high, given that they seem to require abundant food during the active season. To determine their impact on the energy budget of hibernators would constitute an important step in the understanding of telomere dynamics in free-living organisms. Furthermore, studies on the seasonal timing of telomere elongation, particularly on telomerase activity, and its dependency on environmental conditions are needed to obtain a clearer picture of the animals' somatic maintenance capacity.
